# Rheumatoid arthritis reduces the risk of colorectal cancer through immune inflammation mediation

**DOI:** 10.1111/jcmm.18515

**Published:** 2024-07-03

**Authors:** Qifan Li, Liang Zhou, Dan Xia, Jiaqian Wang

**Affiliations:** ^1^ Department of Orthopaedics The Affiliated Suqian First People's Hospital of Nanjing Medical University Suqian China; ^2^ Department of Orthopaedic Lianshui county People's Hospital Huai'an China; ^3^ Department of Respiratory The Affiliated Wuxi Fifth Hospital of Jiangnan University Wuxi China; ^4^ Department of Orthopaedic Zhongshan Hospital, Fudan University Shanghai China

**Keywords:** immune inflammation, malignancy, mendelian randomization, pan‐cancer, rheumatoid arthritis

## Abstract

There is a close relationship between immune‐mediated inflammation and cancer, and there is still controversy over whether rheumatoid arthritis (RA) increases the risk of malignancy. We first used Mendelian randomization (MR) analysis to explore the potential causal relationship between RA and pan‐cancer. And verify the effect of immune‐mediated inflammation on cancer through intermediate MR analysis. Then we extracted the standardized incidence rate of malignancy in RA patients relative to the general population through large‐scale meta‐analysis. Finally, we performed pan‐cancer analysis on the RA related genes obtained from MR analysis. And perform immune related analysis on key genes to reveal the association between RA and malignancy. The MR analysis demonstrated a negative correlation between RA and pan‐cancer (*p* = 0.008). Autoimmune traits were the main mediating variable for the causal relationship between RA and pan‐cancer. Based on the results of the meta‐analysis, we validated that RA reduces the risk of developing colorectal cancer (SIR = 0.69, 95% CI 0.53–0.85). Pan‐cancer analysis also showed that high expression of RA related genes was negatively correlated with colon adenocarcinoma. IL6R was the gene with the highest correlation among them, and its correlation with immune cells was higher in colorectal cancer than in other malignancy. Our MR study provides evidence that RA was associated with reduced risk of colorectal cancer. This effect is caused by immune‐mediated inflammation, with IL6R being a key regulatory gene.

## 1 INTRODUCTION

Malignancy is a systemic disease characterized by chronic inflammation. Whether this inflammation triggers tumour development or helps tumour growth depends on the overall state of the body.^1^ But at the same time, in the development of tumours, the immune system also has strong anti‐tumour properties. Immunotherapy targeting the immune system has completely changed the treatment philosophy for tumours.^2^ By regulating the patient's immune system through immune checkpoint inhibitors such as anti‐PD‐1 and PD‐L1, various types of tumours have been able to achieve long‐term remission.^3^ Rheumatoid arthritis (RA) is a chronic, systemic, inflammatory autoimmune disease characterized by erosive arthritis. In addition to causing joint swelling, pain, stiffness and deformities, it can also damage multiple systems of the body, including the skin, lungs, heart and blood vessels, causing great harm to patients.^4^ The exact cause of RA is not yet clear, and factors such as immunity, infection and genetics are closely related.^5^, ^6^

The stimulation of abnormal chronic inflammation caused by RA, genetic susceptibility and regulation of immune response in RA treatment may all be related to the occurrence of malignant tumours.^7^ Mechanistically speaking, both diseases exhibit abnormal immune activation and inflammatory processes.^8^ In RA patients, a large number of immune cells participate in the autoimmune response, leading to a decrease in immune monitoring ability.^9^ Meanwhile, the production of persistent chronic inflammatory mediators such as interleukin‐6 (IL‐6) increases tumour migration and invasion.^10^ Since Losmaki first described the relationship between RA and malignancy in 1978, there has been a consensus that RA increases the risk of lymphoma.^11^ But it seems that this can only explain the increase in the incidence rate of lymphoma. The relationship between RA and other types of cancer has not yet been elucidated. The research report on the risk of solid cancer in RA patients has yielded inconsistent results, with some solid tumour risks even decreasing.^12^ A nationwide cohort study found that RA patients have a lower risk of gastric, colon and lung cancer compared to the general population.^13^ There is currently no research that can explain this contradictory phenomenon well. The risk of cancer in RA patients may be influenced by many potential confounding factors, including sample size and immunosuppressive therapy against RA.^14^ Thus, more well‐designed methods are needed to evaluate the causal relationship between RA and cancer risk.

Mendelian randomization (MR) has been widely used in causal inference in epidemiology. The main principle of this method is to study the impact of exposure factors on results by using genotypes, avoiding potential confounding factors.^15^ Our aim is to conduct MR analysis to more effectively explore the possible causal relationship between RA and the entire cancer spectrum (pan cancer). Then, further determine this relationship through large‐scale meta‐analysis. Finally, using the database to identify the common molecular mechanisms of these two diseases, in order to find better therapeutic targets for tumour immunity.

## 2 MATERIALS AND METHODS

### 2.1 MR

#### 2.1.1 Data sources

An overview design of our study was shown in Figure S1. The genetic variation data related to RA and pan‐cancer are from the GWAS catalogue and the genetic instrumental variables are composed of related single nucleotide polymorphisms (SNPs) loci. Based on MR analysis of two samples, RA was identified as exposure and the outcome was pan‐cancer. The summary statistics data of RA include 19,234 cases and 61,565 controls (ieu‐a‐833). The genetic instruments summary data of pan cancer include 70,223 cases and 372,016 controls (ieu‐b‐4966). In order to further investigate the causal relationship between immune inflammation and cancer, autoimmune traits served as potential mediators (ebi‐a‐GCST90029015). Determine immune inflammatory effects through MR analysis between potential mediators and outcomes.

#### 2.1.2 Defining genetic instruments and SMR analysis

All genetic instruments for MR selection need to meet three important core conditions to minimize bias.^16^ The genetic instruments are highly correlated with exposure factors (relevance), but not with confounding factors (independence) and can only affect the results through exposure (exclusion‐restriction). Based on the above three assumptions, we obtained genetic variations strongly associated with RA and pan‐cancer (*p* < 5 × 10–8). Secondly, we excluded SNPs of strong linkage disequilibrium (*r* ^2^ < 0.01, kb = 10,000). Finally, evaluate the F‐statistic using a formula to assess potential instrument bias. If the *F*‐statistic >10, it will be included in this study. Perform statistical analysis on the included SNPs through various methods to infer causal relationships.

In order to further identify the regulatory genes of RA in the blood, the summary‐data‐based MR method (SMR) was used to integrate and analyse the GWAS data of RA with the expression quantitative trait loci (eQTLs) from the blood.

#### 2.1.3 Statistical analysis

All analyses were carried out using ‘TwoSampleMR’ and ‘MR‐PRESSO’ packages in R version 4.1.3, with a statistically significant *p* < 0.05. Evaluate the causal relationship between RA and pan cancer through inverse variance weighted (IVW), MR Egger regression, and weighted mode methods. The IVW method, as the main statistical method, weights each instrumental variable by the reciprocal of variance. If there is no intercept, calculate the weighted average of the effective estimates for all instrumental variables. The leave‐one‐out method evaluate the sensitivity of results. By removing SNPs one by one and calculating the effects of merging other units, the effect of a single SNP on outcomes is clarified. We used MR‐PRESSO to detect outliers, and scatter plots indicate whether the results are affected by outliers. Heterogeneity testing tests the differences between instrumental variables, and funnel plots indicate whether the results are robust or heterogeneous.

### 2.2 Meta‐analysis

#### 2.2.1 Search strategy and eligibility criteria

Our work was conducted in accordance with the Preferred Reporting Items for Systematic Reviews and Meta‐Analyses (PRISMA) Statement. We searched for data from PubMed, Embase, and Cochrane Library over the past 20 years (from January 2003 to November 2023). The following search terms were used, alone or in combination: ‘RA’ or ‘autoimmune diseases’ and ‘cancer’ or ‘tumour’ or ‘malignancy’. We did not impose any language restrictions on our search.

We reviewed all the retrieved abstracts and full texts. The research criteria included in this analysis were as follows: (1) Observational studies (including case–control studies, cohort studies and database studies); (2) Reported malignancy outcomes in RA patients and control population; (3) The patient's various types of information are detailed and complete; (4) Malignancy risk was measured by standardized incidence rate (SIRs).

#### 2.2.2 Outcome measures and statistical analysis

We extracted risk data for malignancy from articles that met the inclusion criteria. To prevent bias, we conducted further analysis only when there were more than five studies on each type of malignancy. Malignant tumours include haematological tumours (Hodgkin's lymphoma, non‐Hodgkin's lymphoma and leukaemia). It also includes solid cancer (bladder cancer, brain cancer, breast cancer, cervical cancer, colorectal cancer, oesophageal cancer, kidney cancer, liver cancer, lung cancer, ovarian cancer, pancreatic cancer, prostate cancer, skin cancer, stomach cancer, thyroid cancer) and special types of cancer (melanoma). The specific estimation of the malignancy relative risk was mainly measured by overall and age‐ and sex‐adjusted SIRs relative to the general population.

This work was performed using Stata 17.0 software (StataCorp LP, College Station, TX, USA). We used SIRs combined with 95% confidence interval to evaluate the relative risk of malignancy. We used the random‐effects model (the DerSimonian‐Laird method) for binary data. The I2 statistic was used to assess heterogeneity in the assay. All *p* values <0.05 were considered statistically significant.

### 2.3 Pan‐cancer analysis

#### 2.3.1 Acquisition of pan cancer expression data

We downloaded the standardized pan cancer dataset TCGA TARGET GTEx (PANCAN, *N* = 19,131, *G* = 60,499) from the UCSC database (https://xenabrowser.net/). We extracted the expression data of RA related genes in whole blood obtained by SMR method in various samples. Furthermore, log_2_ (x + 0.001) transformation was applied to each expression value. Then we also excluded cancer species with less than three samples in a single cancer species, and ultimately obtained expression data for 34 cancer species. The R package ‘limma’, ‘ggplot2’ and ‘ggpubr’ were used to compare the expression of RA related genes in different malignancy.

#### 2.3.2 Immune infiltration analysis of key genes in RA

Based on meta‐analysis and pan cancer results, key genes related to RA and malignancy were screened. Then, the ImmuneScore was calculated for 10,180 tumour samples from 44 tumour types by the R package ‘ESTIMATE’. Furthermore, the Pearson's correlation coefficient between genes and immune infiltration scores in a single tumour was calculated using the corr. test function of the R package ‘psych’. And visualize the immune infiltration score and correlation coefficient about the tumour of interest to us.

#### 2.3.3 Correlation analysis of immune genes and cells

We collected 150 immune regulatory genes (41 chemokines, 18 chemokine receptors, 21 MHC related immune genes, 24 immunoinhibitor, 46 immunostimulator) and 60 immune checkpoint pathway genes (24 Inhibitory, 36 Stimulatory) from standardized pan cancer dataset. The correlation coefficient between RA key genes and immune genes was determined by Pearson statistical method. In addition, we utilize the deconvo CIBERSOR method of the R package ‘IOBR’ to evaluate the 22 immune cell infiltration scores of each patient in each tumour based on gene expression. Similarly, the Timer method using the R package ‘IOBR’ was used to evaluate six types of immune cell infiltration scores. And visualize the correlation coefficient between RA key genes and immune cells.

## 3 RESULTS

### 3.1 Causal effect of RA on pan cancer

**FIGURE 1:**
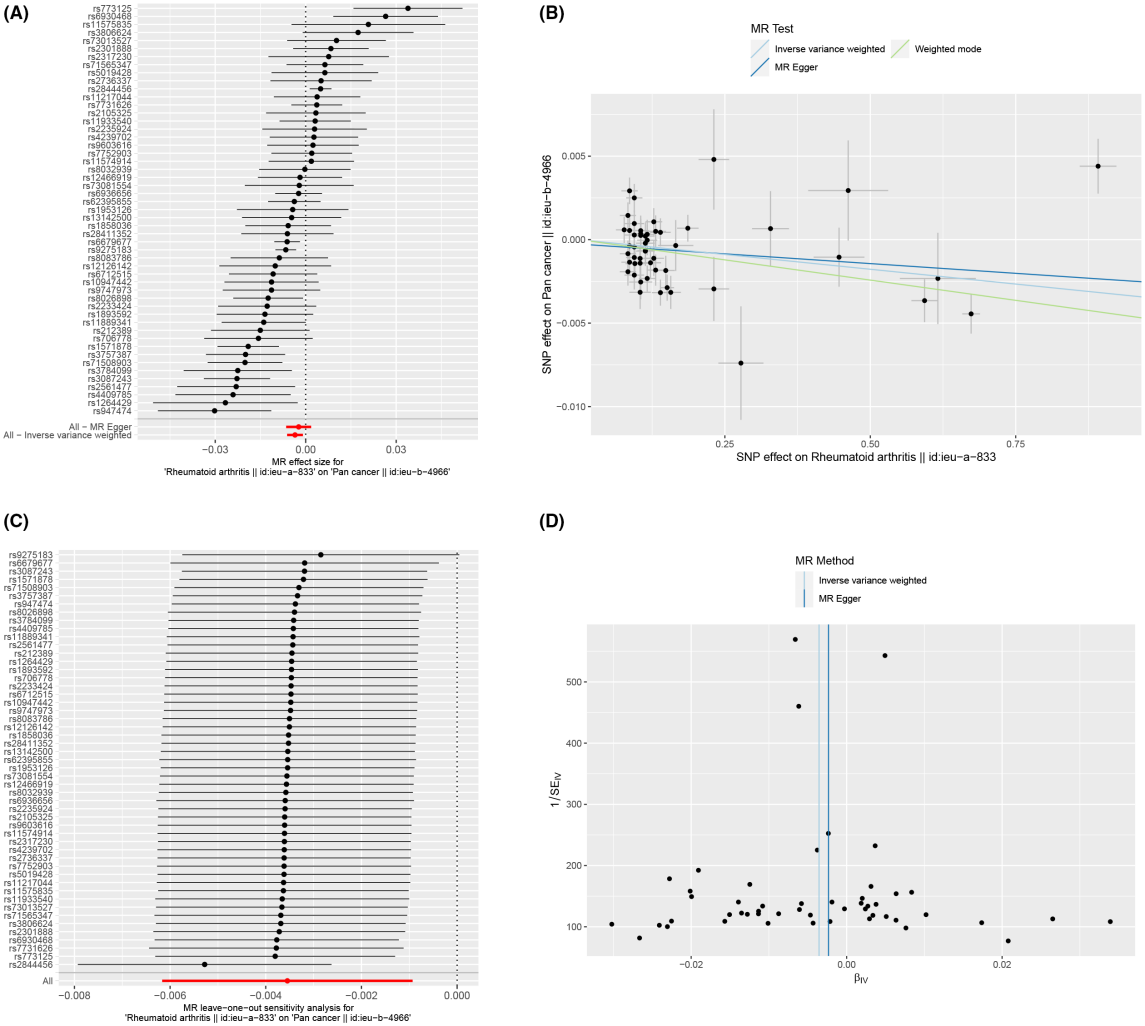
Causal effect of RA on pan‐cancer using different MR methods. (A) Forest plot of using IVW method to comprehensively estimate all SNPs. Horizontal lines represent 95% confidence intervals; (B) Scatter plot of the causal relationships between RA and pan‐cancer. The slope of each line corresponds to the causal estimates for each method; (C) Leave‐one‐out sensitivity analysis for the effect of RA on pan‐cancer. Red point denotes the IVW estimate using all SNPs; (D) Funnel plot represent estimates with all SNPs.

We identified robust SNPs as instrumental variables for RA, respectively, after excluding pleiotropic SNPs (Figure 1A). As shown in Figure 1B, there is a negative causal relationship between RA and pan cancer in MR analysis using the IVW method (OR = 0.996, 95% CI 0.994–0.999, *p* = 0.008). The results of weighted median and weighted mode were also statistically significant, RA may reduce the risk of cancer. The difference between the egger_intercept and zero was not statistically significant (*p* = 0.461), indicating no presence of horizontal pleiotropy among SNPs. In addition, the results of the leave‐one‐out method showed that after gradually removing each SNP, the results of the remaining SNPs were similar to the original results (Figure 1C). The funnel plot also indicates that the results are robust (Figure 1D).

### 3.2 Effect of immunity as a mediator on cancer

**FIGURE 2:**
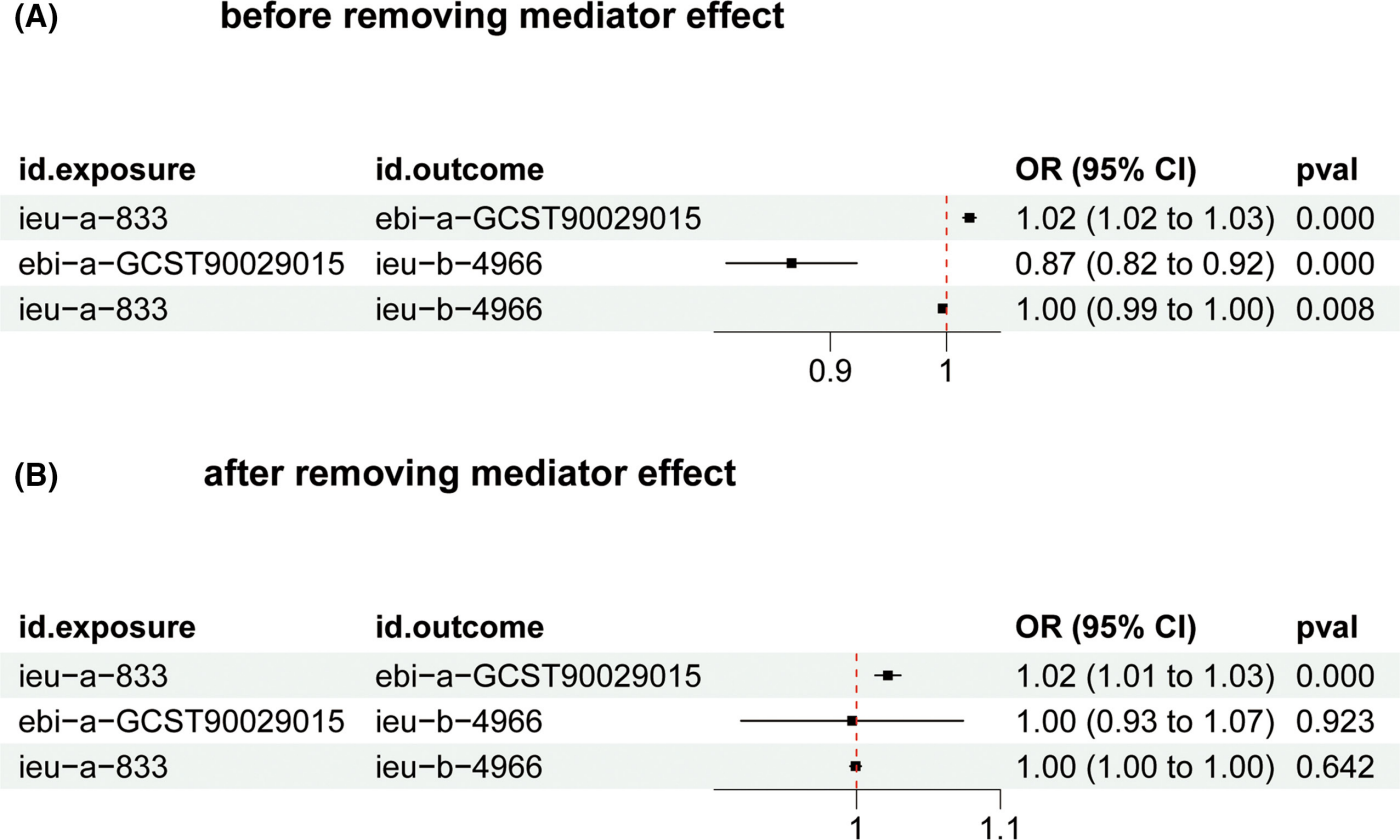
Using MR method to determine the mediator for RA and pan‐cancer. (A) Evaluate the effect of RA on the pan‐cancer before adjusting for the mediator; (B) Evaluate the effect of RA on the pan cancer after adjusting for the mediator. ieu‐a‐833 represent RA; ieu‐b‐4966 represent pan‐cancer; ebi‐a‐GCST90029015 represent autoimmune traits served as potential mediators.

We first conducted MR analysis based on autoimmune traits as exposure and pan cancer as outcome. IVW method showed that autoimmune traits (OR = 0.87, 95% CI 0.82–0.92, *p* = 3.21e‐06) had causal relationship with the decreased risk of pan cancer (Figure 2A). Next, we removed the mediating exposure factors of autoimmune traits. Autoimmune traits (OR = 0.997, 95% CI 0.926–1.07, *p* = 0.923) lost their causal relationship with pan cancer. Most importantly, RA also lost its causal relationship with pan cancer (OR = 0.999, 95% CI 0.996–1.003, *p* = 0.642), confirming that RA reduces cancer risk through immune inflammation mediation (Figure 2B).

### 3.3 Meta‐analysis of malignancy in RA patients

**FIGURE 3:**
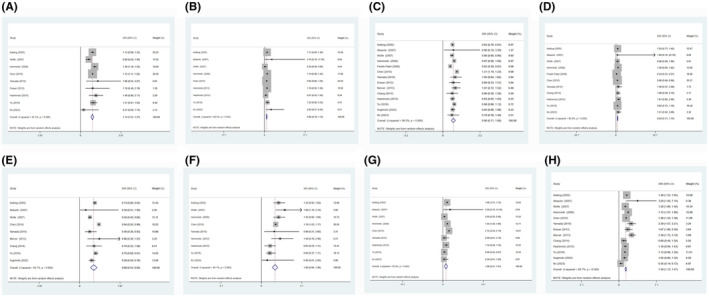
The relative risk of RA patients developing malignancy. (A) Forest plot of bladder cancer risk; (B) Forest plot of brain cancer risk; (C) Forest plot of breast cancer risk; (D) Forest plot of cervical cancer risk; (E) Forest plot of colorectal cancer risk; (F) Forest plot of oesophageal cancer risk; (G) Forest plot of kidney cancer risk; (H) Forest plot of lung cancer risk. Relative risk was measured by the standardized incidence rate (SIRs) relative to the general population.

**FIGURE 4:**
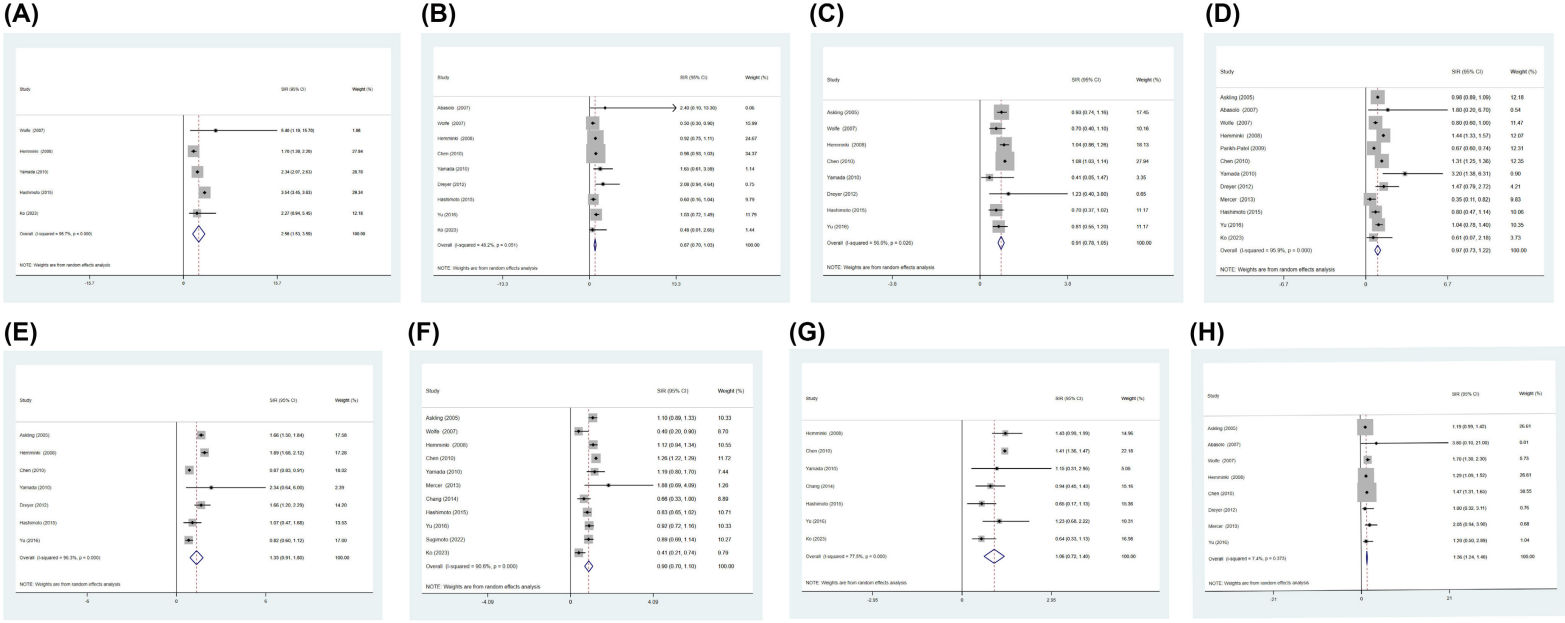
The relative risk of RA patients developing malignancy. (A) Forest plot of liver cancer risk; (B) Forest plot of ovarian cancer risk; (C) Forest plot of pancreatic cancer risk; (D) Forest plot of prostate cancer risk; (E) Forest plot of skin cancer risk; (F) Forest plot of stomach cancer risk; (G) Forest plot of thyroid cancer risk; (H) Forest plot of melanoma risk.

We identified all relevant articles by searching the databases and deleting duplicate ones. After scanning the title and abstract, irrelevant articles are excluded. Subsequently, after reviewing the entire text, the remaining 18 articles were used for further analysis (Table S1). The results showed that the risk of haematological tumours in RA patients was significantly higher than that in the general population (Figure S2). In solid tumours, SIRs and 95% CI of bladder cancer, lung cancer and liver cancer were significantly higher than those of the general population (Figures 3 and 4). In colorectal cancer, it is significantly lower than the general population (SIR = 0.69, 95% CI 0.53–0.85). The risk of colorectal cancer is consistent with the trend obtained from MR analysis.

### 3.4 Expression of IL6R in pan‐cancer

**FIGURE 5:**
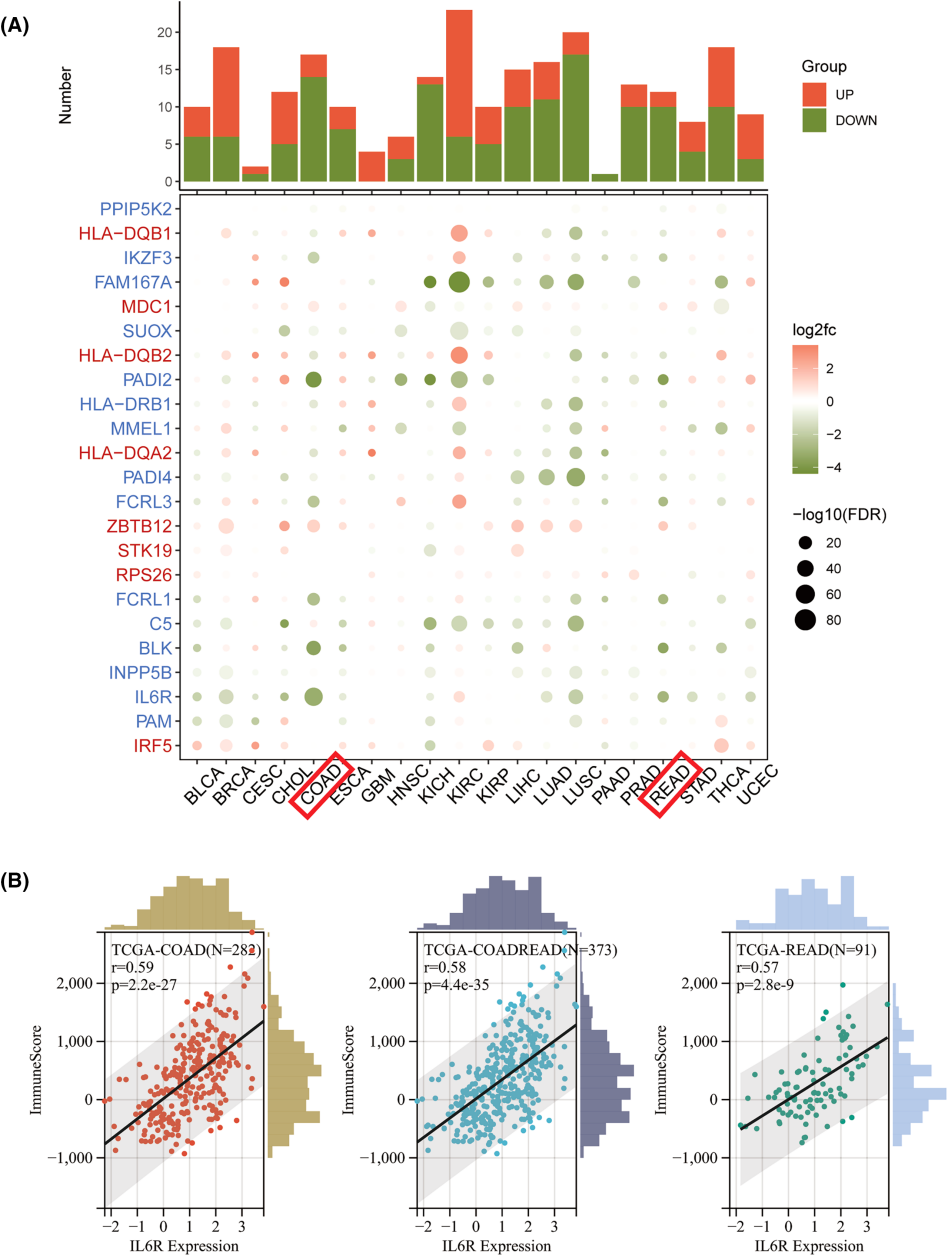
Pan‐cancer analysis of RA pathogenic genes. (A) The correlation and differential expression of RA pathogenic genes with different malignancy; (B) The correlation between RA key gene (IL6R) and immune infiltration in colorectal cancer.

To further verify the negative correlation between RA and colorectal cancer, we obtained the pathogenic genes of RA through SMR analysis (Table S2). By combining and digging into the resources from TCGA and GTEx databases, we compared the expression levels of RA pathogenic genes in pan‐cancer. We found that in most common malignancy, RA pathogenic genes are negatively correlated with them (Figure 5A), including colon adenocarcinoma (COAD), renal chromophobe (KICH), lung squamous cell carcinoma (LUSC) and rectal adenocarcinoma (READ), etc. IL6R has the highest correlation with COAD and READ, and may be a key gene in reducing the risk of colorectal cancer in RA patients.

### 3.5 IL6R correlate with immunity in colorectal cancer

**FIGURE 6:**
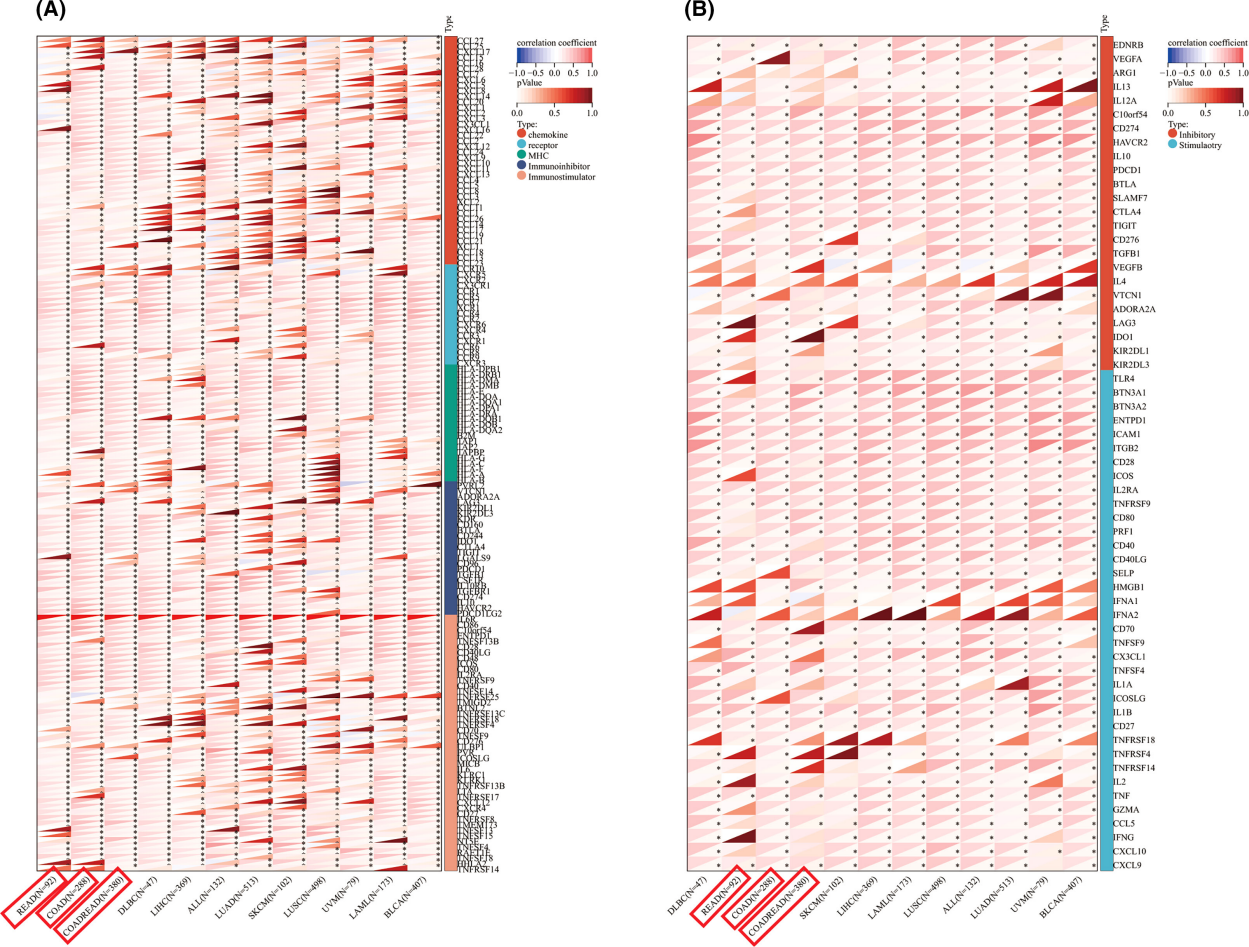
The correlation between IL6R and immune genes in malignancy (statistical significance of malignancy in meta‐analysis). (A) Heatmap of the correlation between IL6R and 150 immune regulatory genes; (B) Heatmap of the correlation between IL6R and 60 immune checkpoint pathway genes.

**FIGURE 7:**
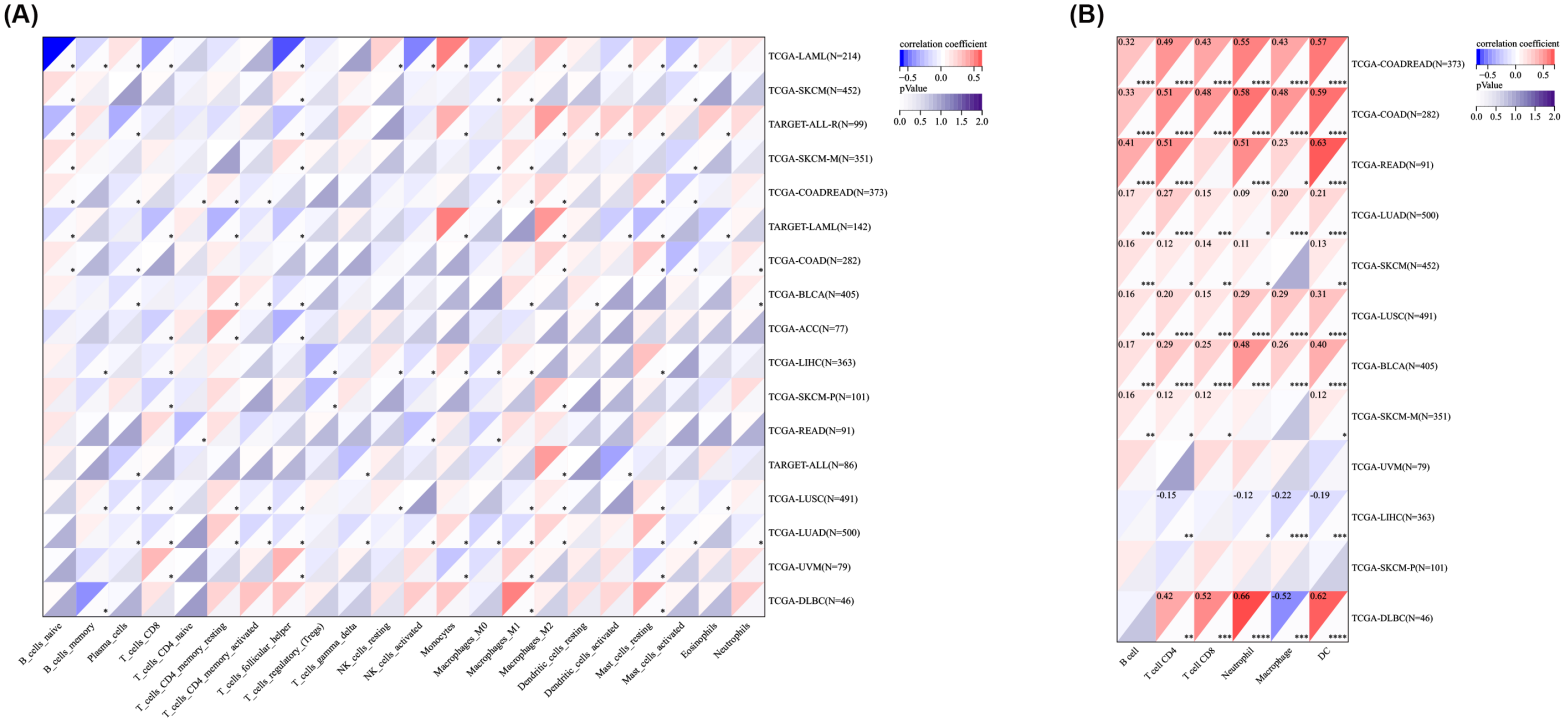
The correlation between IL6R and immune cells in malignancy (statistical significance of malignancy in meta‐analysis). (A) Heatmap of the correlation between IL6R and 22 immune cells using CIBERSOR method; (B) Heatmap of the correlation between IL6R and 6 types of immune cell using Timer method.

Next, for the purpose of exploring the significance of IL6R on the tumour immune microenvironment by investigating the relationship between IL6R and colorectal cancer immunity. According to ImmuneScore, the expression of IL6R in COAD, COADREAD and READ was significantly positively correlated with immune infiltration (Figure 5B). We compared the differential expression of IL6R in relation to immune genes and immune cells between colorectal cancer and (meta‐analysis) high‐risk cancer. Compared to other malignancy, the correlation between IL6R and chemokines and immunostimulators were higher in colorectal cancer (Figure 6A). In the immune checkpoint pathway genes, the differences in correlation between colorectal cancer and other cancer were mainly concentrated in the stimulatory genes (Figure 6B). In addition, the results of immune cell infiltration indicate that the expression of IL6R in colorectal cancer was mainly associated with B cells, macrophage M2 and mast cells (Figure 7A). The comparison results of Timer analysis further indicated that compared to other malignancy, IL6R had a higher correlation with various immune cells in colorectal cancer (Figure 7B).

## 4 DISCUSSION

We identified a negative causal relationship between RA and pan‐cancer through MR analysis, and further validated this negative relationship between RA and the colorectal cancer through meta‐analysis. Multiple research reports have shown that RA patients have a moderately increased overall risk of malignancy compared to the general population.^17^, ^18^ This is contrary to our results, but in the real world, the occurrence and development of malignancy are related to various external factors. The examination of RA patients during hospitalization may lead to an increase in tumour detection rate. Smoking increases the risk of RA and cancer, however, the reported SIR has not been adjusted for smoking and some other influencing factors, so it cannot be ruled out that the increase in SIR is due to an indirect association.^19^ In addition, the use of drugs during the treatment of RA may also interfere with the occurrence of tumours.^20^ Especially Janus kinase (JAK) inhibitors can affect the function of natural killer cells and may have the opposite effect on tumour treatment.^21^ Genetic variation remains stable throughout the lifecycle, allowing MR analysis to be unaffected by causal inversion and confounding factors.^22^ But at the same time, genetic variation also has pleiotropy, which may lead to errors. We combined real‐world meta‐analysis results to make the results more credible.

Immunology has always been an important direction for studying cancer progression, and immune disorders can also lead to the occurrence of tumours. However, there is still controversy over whether RA is related to malignancy. We found that autoimmune traits are important mediating factors between these two diseases, and interventions to reduce immune factors may increase the risk of malignancy in RA patients. So we propose a new perspective that although RA has a pathogenic effect on the human body due to immune disorders leading to the secretion of inflammatory factors and activation of immune cells, this immune activation also has an inhibitory effect on certain malignancy. This may be a reasonable explanation for the reduced risk of colorectal cancer in RA patients. In order to further investigate the specific mechanism, we obtained the pathogenic genes of RA through SMR analysis, including PADI4, HLA‐DRB1 and HLA‐DQB1, which have been confirmed to be related to susceptibility to RA. Then, through pan cancer analysis, we also found that in most common malignant tumours, RA pathogenic genes are negatively correlated with them, with COAD and LUSC having the highest negative correlation. This indirectly confirms the reliability of our research. Among these pathogenic genes, IL6R may play an important role in inhibiting the development of colorectal cancer.

IL6 is a pro‐inflammatory cytokine composed of IL6, receptor IL6R and glycoprotein 130 (gp130), which activates different signalling mechanisms to perform various biochemical functions.^23^ An increasing number of studies have shown that IL‐6 plays an important role in the development of autoimmune diseases, cancer, and COVID‐19.^24^, ^25^ Recently, IL‐6 has been shown to help regulate the development of Th17 cells, which can participate in RA pathogenesis by activating fibroblast like synovial cells (FLS).^26^ IL‐6 also activates STAT3 promoter gene transcription through JAK, promoting cell proliferation and preventing cell apoptosis, leading to tumour formation.^27^ IL‐6 is not only a key factor in the inflammatory pathway of RA, but can also act on different signalling pathways to affect the cell cycle, thereby inducing tumour production. In our study, IL6R showed the highest negative correlation with colorectal cancer. According to the immune infiltration score, IL6R also mediates colorectal cancer through immunity. Satoshi found that circulating IL6 levels are associated with the survival rate of colorectal cancer patients, indicating that IL6 may be a potential target for the treatment of colorectal cancer.^28^

Moreover, there is a significant correlation between the expression level of IL6R and many immune cells, such as T cells, macrophages, neutrophils, DC cells, etc. An interesting and striking observation here is that the correlation between IL6R and immune cells in colorectal cancer and lymphoid neoplasm diffuse large B‐cell lymphoma (DLBC) is significantly higher than that in other malignancy. However, what sets DLBC apart from colorectal cancer was its significant negative correlation with macrophages. Further analysis using the CIBERSOR method revealed that colorectal cancer was associated with macrophage M2, while DLBC was associated with macrophage M1. Tumour‐associated macrophages (TAMs) are frequently associated with poor prognosis in human cancers.^29^ Interestingly, contradictory results have also been reported in several types of human cancers, including colorectal cancer.^30^, ^31^ Studies have shown that TAM infiltration is associated with chemotherapy resistance in colorectal cancer patients. Colorectal cancer conditional macrophages increase the chemical resistance of colorectal cancer and reduce drug‐induced cell apoptosis by secreting IL6, which can be blocked by neutralizing anti‐IL6 antibodies.^32^ Although the causal relationship between macrophages and IL6R in colorectal cancer cannot be determined at present, this lays the foundation for our next exploration of related mechanisms.

## 5 CONCLUSION

In summary, our research findings provide preliminary evidence that there is a negative causal relationship between RA and malignancy. RA reduced the risk of colorectal cancer through immune‐mediated inflammation, and IL6R may be a key regulatory gene among them.

## AUTHOR CONTRIBUTIONS

**Qifan Li:** Funding acquisition (equal); resources (equal); writing – review and editing (equal). **Liang Zhou:** Funding acquisition (equal); methodology (equal); resources (equal); supervision (equal); validation (equal); writing – review and editing (equal). **Dan Xia:** Data curation (lead); investigation (lead); software (equal); visualization (equal); writing – review and editing (lead). **Jiaqian Wang:** Conceptualization (lead); project administration (lead); software (equal); visualization (equal); writing – original draft (lead).

## FUNDING INFORMATION

No benefit in any form have been received or will be received from a commercial party related directly or indirectly to the subject of this article.

## CONFLICT OF INTEREST STATEMENT

The authors declare that they have no conflicts of interest.

## CONSENT

All authors contributed to the article and approved the submitted version.

## Supporting information


Figure S1.

Figure S2.

Tables S1‐S2.

## Data Availability

The data used to support the findings of this study are included within the article.
