# Supplementary figures and images for: Rheumatoid arthritis reduces the risk of colorectal cancer through immune inflammation mediation
Source: J Cell Mol Med. 2024 Jul 3;28(13):e18515. doi: 10.1111/jcmm.18515 (PMC11222658; doi:10.1111/jcmm.18515)

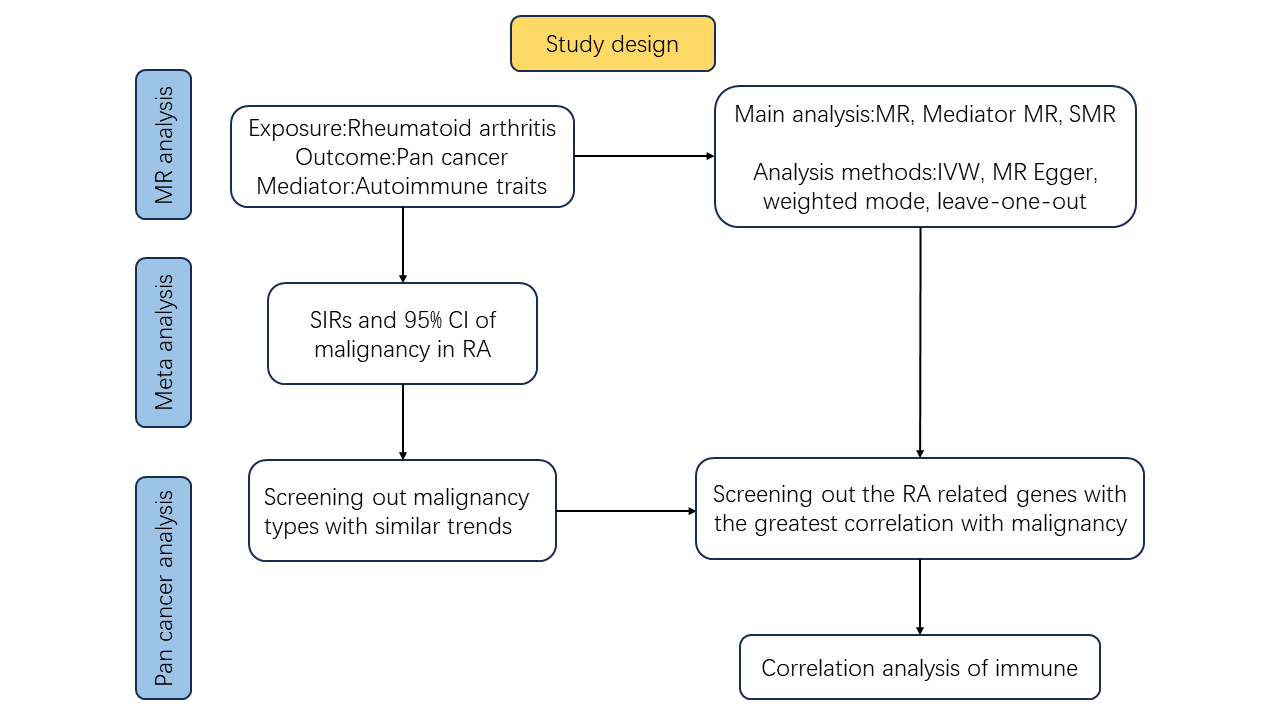

Supplement: Supplementary file 1 — Figure S1. [file JCMM-28-e18515-s003.tif]

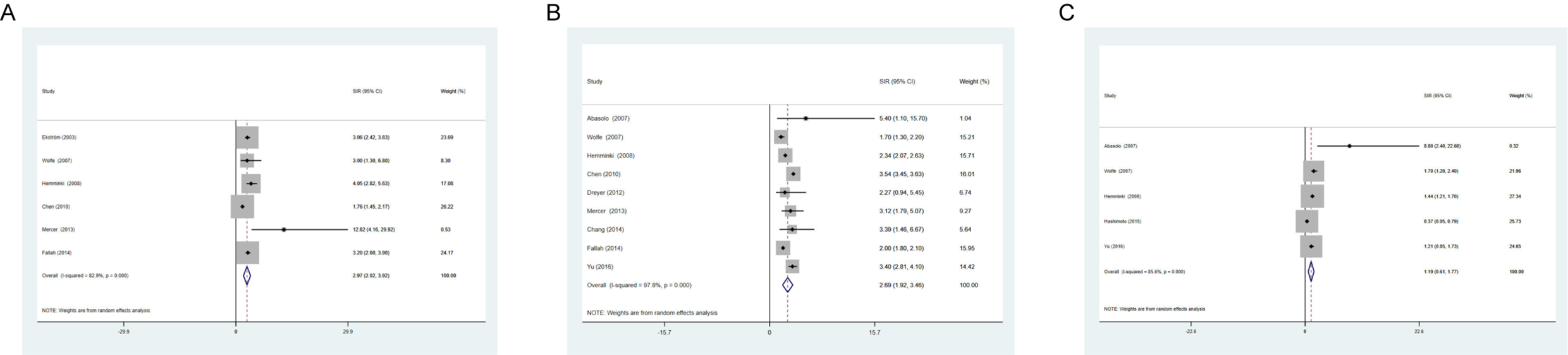

Supplement: Supplementary file 2 — Figure S2. [file JCMM-28-e18515-s002.tif]
