# Supplementary material for: Rheumatoid arthritis reduces the risk of colorectal cancer through immune inflammation mediation
Source: J Cell Mol Med. 2024 Jul 3;28(13):e18515. doi: 10.1111/jcmm.18515 (PMC11222658; doi:10.1111/jcmm.18515)
Supplement: Supplementary file 3 — Tables S1‐S2. [file JCMM-28-e18515-s001.docx]

**TABLE S1** Trials included in the meta-analysis of malignancy patients in RA

| Author | Year | Country | RA | | Outcome cancer |
| --- | --- | --- | --- | --- | --- |
|  |  |  | Cancer number | Total number |  |
| Ekstrom | 2003 | Sweden | 535 | 76527 | 17 |
| Askling | 2005 | Sweden | 3379 | 53067 | 1,2,3,4,5,6,7,8,11,12,13,14,16 |
| Abasolo | 2007 | Spain | 25 | 789 | 2,3,4,5,7,8,10,12,16,18,19 |
| Wolfe | 2007 | America | 543 | 13869 | 1,2,3,4,5,6,7,8,9,10,11,12,14,16,17,18,19 |
| Hemminki | 2008 | Sweden | 4366 | 42262 | 1,2,3,4,6,7,8,9,10,11,12,13,14,15,16,17,18,19 |
| Parikh-Patel | 2009 | America | 5533 | 84475 | 3,4,12 |
| Chen | 2010 | China | 935 | 23644 | 1,2,3,4,5,6,7,8,10,11,12,13,14,15,16,17,18 |
| Yamada | 2010 | Japan | 173 | 7566 | 1,3,4,5,6,7,8,9,10,11,12,13,14,15 |
| Dreyer | 2012 | Denmark | 128 | 3812 | 1,3,8,10,11,12,13,16,18 |
| Hemminki | 2013 | Sweden | 158 | 731954 | 2,6 |
| Mercer | 2013 | Britain | 182 | 3771 | 3,5,8,12,14,16,17,18 |
| Chang | 2014 | South Korea | 106 | 2104 | 3,4,5,8,14,15,18 |
| Fallah | 2014 | Sweden | 698 | 125117 | 18 |
| Fallah | 2014 | Sweden | 93 | 1212967 | 17 |
| Hashimoto | 2015 | Japan | 559 | 66593 | 1,2,3,4,6,7,8,9,10,11,12,13,14,15,19 |
| Yu | 2016 | China | 1604 | 35182 | 1,2,3,4,5,6,7,8,10,11,12,13,14,15,16,18,19 |
| Sugimoto | 2022 | Japan | 507 | 11299 | 3,5,8,14 |
| Ko | 2023 | South Korea | 148 | 5023 | 1,2,3,4,6,7,8,9,10,12,14,15 |

Outcome cancer: 1 represents Bladder cancer; 2 represents Brain cancer; 3 represents Breast cancer; 4 represents Cervical cancer; 5 represents Colorectal cancer; 6 represents Esophagus cancer; 7 represents Kidney cancer; 8 represents Lung cancer; 9 represents Live cancer; 10 represents Ovarian cancer; 11 represents Pancreatic cancer; 12 represents Prostate cancer; 13 represents Skin cancer; 14 represents Stomach cancer; 15 represents Thyroid cancer; 16 represents Melanoma;17 represents Hodgkin’s lymphoma; 18 represents Non-Hodgkin’s lymphoma; 19 represents Leukemia.

**TABLE S2** Summary-data based MR analysis results

| probeID | Gene | topSNP | A1 | A2 | b_SMR | se_SMR | p_SMR | tissue |
| --- | --- | --- | --- | --- | --- | --- | --- | --- |
| ENSG00000238164 | RP3-395M20.8 | rs876938 | C | G | 0.633779 | 0.13172 | 1.50E-06 | Whole_Blood |
| ENSG00000157870 | FAM213B | rs876938 | C | G | 0.449289 | 0.0816285 | 3.71E-08 | Whole_Blood |
| ENSG00000142606 | MMEL1 | rs876938 | C | G | 0.200633 | 0.0323414 | 5.52E-10 | Whole_Blood |
| ENSG00000215912 | TTC34 | rs876938 | C | G | -0.471728 | 0.096024 | 8.99E-07 | Whole_Blood |
| ENSG00000117115 | PADI2 | rs2235924 | A | G | 0.164348 | 0.0302317 | 5.44E-08 | Whole_Blood |
| ENSG00000159339 | PADI4 | rs2301888 | A | G | -1.06841 | 0.224252 | 1.89E-06 | Whole_Blood |
| ENSG00000204084 | INPP5B | rs28411352 | T | C | 0.328329 | 0.0561175 | 4.89E-09 | Whole_Blood |
| ENSG00000160712 | IL6R | rs12126142 | A | G | 0.952977 | 0.226679 | 2.62E-05 | Whole_Blood |
| ENSG00000160856 | FCRL3 | rs2317230 | T | G | 0.276818 | 0.0555706 | 6.31E-07 | Whole_Blood |
| ENSG00000163534 | FCRL1 | rs2317230 | T | G | -0.421111 | 0.10474 | 5.81E-05 | Whole_Blood |
| ENSG00000145730 | PAM | rs2561477 | A | G | -0.301101 | 0.0681491 | 9.95E-06 | Whole_Blood |
| ENSG00000145725 | PPIP5K2 | rs2561477 | A | G | 0.457894 | 0.0884472 | 2.25E-07 | Whole_Blood |
| ENSG00000137337 | MDC1 | rs1264429 | C | T | -0.710938 | 0.139956 | 3.78E-07 | Whole_Blood |
| ENSG00000198563 | DDX39B | rs2844456 | C | T | -2.21565 | 0.377961 | 4.57E-09 | Whole_Blood |
| ENSG00000204366 | ZBTB12 | rs6936656 | T | C | -0.665353 | 0.109263 | 1.13E-09 | Whole_Blood |
| ENSG00000204344 | STK19 | rs71565347 | A | G | 0.915069 | 0.19395 | 2.38E-06 | Whole_Blood |
| ENSG00000250535 | STK19P | rs71565347 | A | G | -0.287075 | 0.0518789 | 3.14E-08 | Whole_Blood |
| ENSG00000196126 | HLA-DRB1 | rs9275183 | G | A | -4.36888 | 0.738511 | 3.30E-09 | Whole_Blood |
| ENSG00000179344 | HLA-DQB1 | rs9275183 | G | A | -1.84314 | 0.255613 | 5.57E-13 | Whole_Blood |
| ENSG00000237541 | HLA-DQA2 | rs9275183 | G | A | 0.775424 | 0.0642432 | 1.52E-33 | Whole_Blood |
| ENSG00000232629 | HLA-DQB2 | rs9275183 | G | A | 1.45929 | 0.20486 | 1.05E-12 | Whole_Blood |
| ENSG00000128604 | IRF5 | rs3757387 | C | T | 0.288398 | 0.0382285 | 4.56E-14 | Whole_Blood |
| ENSG00000154319 | FAM167A | rs2736337 | C | T | 0.141012 | 0.0208944 | 1.49E-11 | Whole_Blood |
| ENSG00000136573 | BLK | rs2736337 | C | T | -0.459418 | 0.0754829 | 1.15E-09 | Whole_Blood |
| ENSG00000255354 | RP11-148O21.2 | rs2736337 | C | T | -0.594642 | 0.124828 | 1.90E-06 | Whole_Blood |
| ENSG00000226752 | PSMD5-AS1 | rs1953126 | C | T | 0.159049 | 0.0285625 | 2.57E-08 | Whole_Blood |
| ENSG00000106804 | C5 | rs1953126 | C | T | -0.452384 | 0.11016 | 4.01E-05 | Whole_Blood |
| ENSG00000139531 | SUOX | rs773125 | G | A | 0.409985 | 0.0784013 | 1.70E-07 | Whole_Blood |
| ENSG00000197728 | RPS26 | rs773125 | G | A | -0.089921 | 0.0151384 | 2.85E-09 | Whole_Blood |
| ENSG00000161405 | IKZF3 | rs9747973 | T | C | 1.06725 | 0.222343 | 1.59E-06 | Whole_Blood |
| ENSG00000073605 | GSDMB | rs9747973 | T | C | -0.296434 | 0.0475941 | 4.71E-10 | Whole_Blood |
| ENSG00000172057 | ORMDL3 | rs9747973 | T | C | -0.257973 | 0.0424416 | 1.21E-09 | Whole_Blood |
| ENSG00000160185 | UBASH3A | rs1893592 | C | A | -0.411498 | 0.0699181 | 3.97E-09 | Whole_Blood |

**Figure legends:**

**Figure S1.** Flow diagram of the study design.

**Figure S2.** The relative risk of RA patients developing hematological tumors. (A) Forest plot of Hodgkin's lymphoma risk; (B) Forest plot of non-Hodgkin's lymphoma risk; (C) Forest plot of leukemia risk.

**References**

1. Ekström, K, Hjalgrim, H, Brandt, L, et al. Risk of malignant lymphomas in patients with rheumatoid arthritis and in their first-degree relatives. ARTHRITIS RHEUM-US. 2003; 48 ARTHRITIS RHEUM-US. doi: 10.1002/art.10939
2. Askling, J, Fored, CM, Brandt, L, et al. Risks of solid cancers in patients with rheumatoid arthritis and after treatment with tumour necrosis factor antagonists. ANN RHEUM DIS. 2005; 64 ANN RHEUM DIS. doi: 10.1136/ard.2004.033993
3. Abásolo, L, Júdez, E, Descalzo, MA, et al. Cancer in rheumatoid arthritis: occurrence, mortality, and associated factors in a South European population. SEMIN ARTHRITIS RHEU. 2008; 37 SEMIN ARTHRITIS RHEU. doi: 10.1016/j.semarthrit.2007.08.006
4. Wolfe, F, Michaud, K. Biologic treatment of rheumatoid arthritis and the risk of malignancy: analyses from a large US observational study. ARTHRITIS RHEUM-US. 2007; 56 (9): 2886-95. doi: 10.1002/art.22864
5. Hemminki, K, Li, X, Sundquist, K, et al. Cancer risk in hospitalized rheumatoid arthritis patients. RHEUMATOLOGY. 2008; 47 (5): 698-701. doi: 10.1093/rheumatology/ken130
6. Parikh-Patel, A, White, RH, Allen, M, et al. Risk of cancer among rheumatoid arthritis patients in California. CANCER CAUSE CONTROL. 2009; 20 (6): 1001-10. doi: 10.1007/s10552-009-9298-y
7. Chen, YJ, Chang, YT, Wang, CB, et al. The risk of cancer in patients with rheumatoid arthritis: a nationwide cohort study in Taiwan. ARTHRITIS RHEUM-US. 2011; 63 (2): 352-8. doi: 10.1002/art.30134
8. Yamada, T, Nakajima, A, Inoue, E, et al. Incidence of malignancy in Japanese patients with rheumatoid arthritis. RHEUMATOL INT. 2010; 31 (11): 1487-92. doi: 10.1007/s00296-010-1524-0
9. Dreyer, L, Mellemkjær, L, Andersen, AR, et al. Incidences of overall and site specific cancers in TNFα inhibitor treated patients with rheumatoid arthritis and other arthritides - a follow-up study from the DANBIO Registry. ANN RHEUM DIS. 2012; 72 (1): 79-82. doi: 10.1136/annrheumdis-2012-201969
10. Hemminki, K, Liu, X, Försti, A, et al. Subsequent brain tumors in patients with autoimmune disease. NEURO-ONCOLOGY. 2013; 15 (9): 1142-50. doi: 10.1093/neuonc/not070
11. Mercer, LK, Davies, R, Galloway, JB, et al. Risk of cancer in patients receiving non-biologic disease-modifying therapy for rheumatoid arthritis compared with the UK general population. RHEUMATOLOGY. 2013; 52 (1): 91-8. doi: 10.1093/rheumatology/kes350
12. Chang, SH, Park, JK, Lee, YJ, et al. Comparison of cancer incidence among patients with rheumatic disease: a retrospective cohort study. ARTHRITIS RES THER. 2014; 16 (4): 428. doi: 10.1186/s13075-014-0428-x
13. Fallah, M, Liu, X, Ji, J, et al. Hodgkin lymphoma after autoimmune diseases by age at diagnosis and histological subtype. ANN ONCOL. 2014; 25 (7): 1397-1404. doi: 10.1093/annonc/mdu144
14. Fallah, M, Liu, X, Ji, J, et al. Autoimmune diseases associated with non-Hodgkin lymphoma: a nationwide cohort study. ANN ONCOL. 2014; 25 (10): 2025-2030. doi: 10.1093/annonc/mdu365
15. Hashimoto, A, Chiba, N, Tsuno, H, et al. Incidence of malignancy and the risk of lymphoma in Japanese patients with rheumatoid arthritis compared to the general population. J RHEUMATOL. 2015; 42 (4): 564-71. doi: 10.3899/jrheum.140533
16. Yu, KH, Kuo, CF, Huang, LH, et al. Cancer Risk in Patients With Inflammatory Systemic Autoimmune Rheumatic Diseases: A Nationwide Population-Based Dynamic Cohort Study in Taiwan. MEDICINE. 2016; 95 (18): e3540. doi: 10.1097/MD.0000000000003540
17. Sugimoto, N, Tanaka, E, Inoue, E, et al. Trends in risks of malignancies in Japanese patients with rheumatoid arthritis: Analyses from a 14-year observation of the IORRA cohort. MOD RHEUMATOL. 2023; 33 (4): 715-722. doi: 10.1093/mr/roac085
18. Ko, KM, Moon, SJ. Prevalence, incidence, and risk factors of malignancy in patients with rheumatoid arthritis: a nationwide cohort study from Korea. KOREAN J INTERN MED. 2021; 38 (1): 113-124. doi: 10.3904/kjim.2021.146
